# Hindlimb Suspension and SPE-Like Radiation Impairs Clearance of Bacterial Infections

**DOI:** 10.1371/journal.pone.0085665

**Published:** 2014-01-15

**Authors:** Minghong Li, Veronica Holmes, Yu Zhou, Houping Ni, Jenine K. Sanzari, Ann R. Kennedy, Drew Weissman

**Affiliations:** 1 Division of Infectious Diseases, Department of Medicine, Perelman School of Medicine, University of Pennsylvania, Philadelphia, Pennsylvania, United States of America; 2 Department of Radiation Oncology, Perelman School of Medicine, University of Pennsylvania, Philadelphia, Pennsylvania, United States of America; Medical University of Graz, Austria

## Abstract

A major risk of extended space travel is the combined effects of weightlessness and radiation exposure on the immune system. In this study, we used the hindlimb suspension model of microgravity that includes the other space stressors, situational and confinement stress and alterations in food intake, and solar particle event (SPE)-like radiation to measure the combined effects on the ability to control bacterial infections. A massive increase in morbidity and decrease in the ability to control bacterial growth was observed using 2 different types of bacteria delivered by systemic and pulmonary routes in 3 different strains of mice. These data suggest that an astronaut exposed to a strong SPE during extended space travel is at increased risk for the development of infections that could potentially be severe and interfere with mission success and astronaut health.

## Introduction

A major risk of extended space travel is the combined effects of weightlessness and radiation exposure on the immune system. Space flight has been shown to alter immune responses, certain of which could lead to potentially detrimental pathology. The causal factors include the stress due to high-demand activities and confinement [1], [2], microgravity [3], [4], [5], diet [6], [7], non-load bearing status [8], and radiation [9], [10], [11]. The consistent effects on the immune system observed during space travel, thus far, are a reduction in peripheral T-cell counts; a decrease in NK cell number and functionality [12], [13]; decreases, sometimes severe, in cell-mediated immunity with altered cytokine production [13], [14]; and normal levels of serum immunoglobulins [13]. An increased susceptibility to infection under space flight conditions has also been observed [15], [16], [17], [18], [19], [20], [21]. Thus, the main concerns of an impaired immune system in the closed environment of a spacecraft is the altered ability to control bacterial, fungal, viral, and parasitic invasions [9], [12], [13], [22] and the loss of immunosurveillance leading to tumor growth [23].

A number of latent viruses have been observed to become reactivated during space flight. Varicella Zoster virus (VZV) was found in 30% of 200 samples taken during and after flight, while no VZV reactivation was found in ground controls [16]. Urinary shedding of cytomegalovirus was observed during a 9-day space flight [17], and increased levels of Epstein Barr virus DNA were observed during and after space flight [17], [18]. Bacterial infections have occurred during and soon after space flight. Fifteen of 29 Apollo astronauts contracted bacterial or viral infections either during their missions or within a week of returning. A urinary tract infection with *Pseudomonas aeruginosa* was documented after an Apollo mission [15]. Twenty-nine infectious disease incidents, including fungal infection, flu-like illness, urinary tract infection, viral gastroenteritis, and skin infection have been documented in approximately 742 crew members on 106 space shuttle flights [21]. Animal models of space flight have demonstrated a reduced ability to clear infections by *Klebsiella pneumoniae* [24] and *Pseudomonas aeruginosa* [25].

Exposure to higher radiation doses than those present in the Earth environment is considered to be a major hazard for astronauts during space travel. The radiation doses and dose rates during space travel are strongly dependent on a number of parameters, which include altitude and shielding [26]. At the International Space Station (ISS), which is in low earth orbit (LEO), there are two major sources of radiation to which astronauts are exposed; these include galactic cosmic rays (GCR) and trapped-belt radiation (mostly protons). In addition, solar particle event (SPE) radiation also contributes to the LEO radiation environment.

Occasional periods of high energy particle flux from the sun, known as SPEs, can result in sufficiently high radiation doses to cause the symptoms of acute radiation sickness (ARS) in astronauts outside the protection of Earth's geomagnetic field, as well as threaten mission success [27]. SPEs consist primarily of relatively low energy protons, with a minor contribution of electrons, alpha particles and heavier particles of high charge and energy, known as HZE particles [28]. SPEs occur quite often (with frequencies of up to 3 per day [29]), but it is difficult to predict their onset and size; the expected overall frequency, however, is strongly influenced by the solar cycle, with a general increase in SPE occurrence with increasing solar activity [29]. SPEs can last from several hours to several weeks [29], [30], [31].

Astronaut extravehicular activity (EVA) is common in NASA missions, but the number of EVAs per mission is highly variable. EVAs performed as part of ISS activities involve relatively low space radiation doses, due to the shielding from the earth's magnetic field, as described above, and the shielding from their spacesuits [27]. Following notice that an SPE is occurring, or is imminent, it would take astronauts performing EVA on the ISS very little time to return to the relative protection of ISS, so their exposure to SPE radiation would be minimal. The radiation doses that astronauts could receive from exposure to SPE radiation could be far greater during future exploration class missions planned by NASA; these mission are expected to involve spaceflight outside LEO in deep space for extended periods of time, as well as lengthy EVAs on the surface of planets or other celestial bodies. Examples of such exploration class missions would include the voyage to Mars, which is expected to last approximately 3 years; some of the planned astronaut activities will involve “extensive” EVAs [32].

Radiation doses have been estimated for astronauts during EVAs or inside the spacecraft from modeling three different historical SPEs (August, 1972, October, 1989 and September, 1989) [27]. Based on model assumptions, it is expected that exposure to these historical events would cause moderate early health effects in crew members both inside the spacecraft or during EVAs, if medical countermeasure tactics are not provided [27]. Exposure to SPE radiation results in an inhomogeneous dose distribution, with high skin doses and lower internal organ doses, since the majority of the protons in SPEs have kinetic energies of 100 MeV or less, which have limited penetrating abilities [28]. SPEs have been characterized as soft or hard, with a hard SPE involving significant levels of high energy protons capable of deep penetration into mammalian tissues and soft SPEs involving primarily lower energy protons with relatively low penetrating ability [33]. Using the historical August 1972 SPE, the estimated doses that an astronaut could receive would be 3215 cGy to the skin and 138.4 cGy-equivalent (cGy-eq) to the blood forming organs during EVA [27]. It has been estimated that the highest dose to internal organs from exposure to SPE radiation would be 2 Gy [34].

Using the hindlimb suspension model of microgravity that includes; situational and confinement stress, fluid shifts, alterations to fluid and food intake, and non-load bearing status combined with SPE-like radiation, the effects on the ability of 3 different strains of mice to respond to and clear 2 different types of bacteria using 2 different sites of challenge were investigated. Both hindlimb suspension and SPE-like proton or reference gamma radiation impaired the ability to control and clear infections and the combination, as could occur during an extended mission outside the Earth's magnetic field, resulted in at least an additive effect resulting in high levels of morbidity.

## Materials and Methods

### Humane care and use of animals

This study was carried out in strict accordance with the recommendations in the Guide for the Care and Use of Laboratory Animals of the National Institutes of Health. The protocol was approved by the Institutional Animal Care and Use Committee (Assurance # A3079-01) of the University of Pennsylvania. Facilities housing the animals involved were accredited by the Association for Assessment and Accreditation of Laboratory Animal Care, International (AAALAC) and inspected regularly by the U.S. Department of Agriculture (USDA).

### Irradiation of mice

Male and female outbred ICR, Balb/c, or C3H/HeN mice 5–6 weeks of age, were obtained from Harlan Laboratories. For irradiation, the mice were placed in aerated plastic chambers (AMAC #530C) with dimensions of 7.30 cm×4.13 cm×4.13 cm, and irradiated with whole body gamma or proton radiation at a dose of 1–2 Gy. The chambers allowed the mice to easily turn around (reverse nose to tail direction). Gamma radiation was delivered using a ^60^Co source (Eldorado Model ‘G’ machine, Atomic Energy of Canada Ltd, Commercial Products Division). The gamma-radiation exposures were delivered in a single fraction at a 50 cGy/min dose rate. The proton beam was produced by the University of Pennsylvania (Penn) IBA cyclotron system, and the mice were irradiated with protons at the Roberts Proton Therapy Center. The 230 MeV proton beam extracted from the cyclotron was degraded using the energy selection system to a nominal energy of 151 MeV or range of 16 cm water equivalent thickness (WET). The degraded beam was delivered in double scattering mode with a spread out Bragg peak (SOBP) modulation width of 5 cm. A 23 cm×17 cm opening in the tungsten multi-leaf collimator (MLC) shaped the beam to a useable field size (>95% of maximum within the flat region) of 20.6 cm×17 cm at the gantry isocenter. The mice enclosures were arranged so that they formed a 14.2 cm×16.4 cm target area. The center of the enclosure array was placed at the gantry isocenter with an additional 11 cm WET of solid water plastic (Gammex Inc.) placed directly in front of the enclosure array, further degrading the proton beam energy to approximately 74 MeV or a range of ∼4.5 cm WET. 5 cm WET of solid water plastic was placed directly behind the enclosure array. The mouse enclosures were irradiated with a range of proton energies forming the uniformly modulated dose region of the SOBP. The dose averaged LET of the proton radiation is low (<10 keV/µm) within the mid-SOBP where the mice are located, and rises to higher LET (>10 keV/µm) towards the downstream edge of the SOBP, which lies beyond the mice enclosures [35]. Dosimetry verification was performed before the irradiations with a 2D ion chamber array (I'm*RT* MatriXX, IBA dosimetry) placed at a depth of 13.3 cm WET. Sham irradiated control mice were also restrained in custom designed plastic chambers and transported to the proton irradiation facility, but were not subjected to irradiation. All mice, irradiated and sham irradiated, were maintained in the plastic chambers for the same period of time corresponding to 2 Gy of irradiation. The proton radiation exposures were delivered in a single fraction at a dose rate of 50 cGy/min. Mouse proton irradiations at the Roberts Proton Therapy Center have been described previously [36], [37].

### Hindlimb unloading

Mice were hindlimb unloaded as described previously [38]. Individual mice were suspended by the tail at 15° head-down tilt with no load bearing on the hindlimbs. Access to food and water was ensured using both water bottles and gel packs and food distributed around the floor of the cage. Animals demonstrated no adverse effects or pronounced weight loss. Groups of 10 mice per treatment per experiment were used due to hindlimb suspension cage limitations.

**Figure 1:**
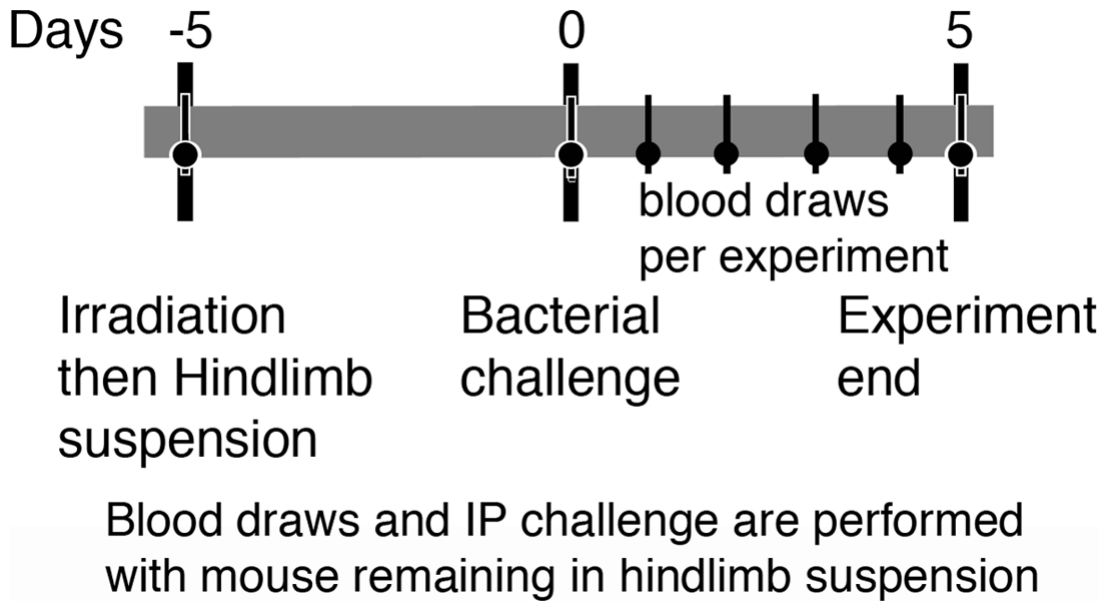
Diagram of experimental procedure. Groups of mice received radiation or not and were then hindlimb suspended or not (black bar). Five days later, all groups were challenged with bacteria and followed for 5 days. Depending on the experiment, blood was obtained with mice remaining in suspension at various times pre and post bacterial challenge (inverted lollipop).

Groups of 10 mice, either male or female, per treatment were used. Mice were irradiated and then placed in hindlimb suspension. Five days later, animals were challenged with bacteria (Figure 1). All blood draws and intraperitoneal injections were performed while mice remained in hindlimb suspension without anesthesia. Inhalation of bacteria required the mice to be anesthetized out of hindlimb suspension.

Blood was obtained at various times before, during, and after hindlimb suspension and/or irradiation by cheek lancet. Blood was obtained without anesthesia and with the animal remaining in hindlimb suspension at the same time (9 AM) for all experiments. This allowed us to rapidly obtain small quantities of blood with minimal additional stress to the mice. Serum was separated by centrifugation at 4,000 RPM for 4 min in an Eppendorf microfuge and frozen at −80°C.

### Systemic infection

The systemic infection model in mice used modifications of a previously described method [39]. *Pseudomonas aeruginosa* (Schroeter) Migula (ATCC, 27853) was cultured overnight in brain heart infusion broth and bacterial cultures were diluted to 10^5^ CFU/µL. Initial studies determined in untreated control mice the maximum amount of bacteria that allowed clearance from the blood and minimal morbidity over 5 days. The relationship between culture optical density (OD) at 600 nm (WPA CO 8000 Cell Density Meter, Biochrom) and counts of bacterial capable of forming colonies after plating (CFU/µl) was ascertained and used to determine the challenge inoculum. The challenge inoculum was spread on agar plates to confirm the challenge dose. Three hundred µls of diluted bacterial suspension were inoculated intraperitoneally (IP) into mice (18–22 g) that were observed for 5 days. Blood was obtained daily for granulocyte counts or at 3 days after the mice were challenged for CFU counting. Changes in the number of animals that did not clear bacteria or experienced an increase in morbidity (Table 1) or death were calculated.

**Table 1:** Assessing morbidity.

Score	Morbidity level	Characteristics
1	No indication of morbidity	Normal, well groomed, alert, active, good condition, asleep or calm, normal appetite
2	Mild morbidity	Not well groomed, awkward gait, slightly hunched, mildly agitated
3	Moderate morbidity	Rough hair coat, squinted eyes, moves slowly, walks hunched and/or slowly, depressed or moderately agitated, slight dehydration, pruritic, restless, uncomfortable, not eating or drinking
4	Severe morbidity	Very rough hair coat, eyes sunken (severe dehydration), slow to move or nonresponsive when coaxed, hunched, dyspnea, self-mutilating, violent reaction to stimuli or when approached

### Respiratory tract infection model

The maximum number of bacteria that allowed mice to clear a respiratory challenge with *Klebsiella pneumoniae* (Schroeter) Trevisan (ATCC, 43816) with minimal morbidity over 5 days was determined and used. Groups of 10 mice per treatment were exposed to bacteria by placing 20 µl of bacteria in PBS into the nares during anesthesia and allowing the mice to inhale. Three days later, blood was obtained to measure CFUs and 5 days after infection, blood and lung tissue was obtained, homogenized, and plated as described below.

### CFU determination

Blood and lung homogenates were obtained. Tenfold serial dilutions of each sample were made in tubes containing 0.9 ml of cold PBS prior to plating 0.1 ml from selected dilutions onto MacConkey agar plates. Plates were incubated for 1 day at 37°C to determine the number of CFU.

### Serum analyte determinations

Total corticosterone levels were analyzed using the Cortisol EIA Kit (cat. # 500360, Caymen Chemical Co.) on serum diluted 1∶20 with PBS. Serum samples were first treated with Steroid Displacement Reagent (1∶100) (Enzo Life Sciences), diluted with PBS, and centrifuging through 96-well filtration plates with a 10 kDa MW cutoff (Millipore Ultracel-10 Filtration Plates) to separate immunoglobulins from free corticosterone.

### Blood cell analysis

Whole blood (50 µl) was stained for CD14 (monocytes) (clone rmC5-3, BD Biosciences), CD11b (activation - monocytes, macrophages, granulocytes, and NK cells) (clone M1/70, BD Biosciences), F4-80 (monocytes) (clone BM8, Biolegend), and Ly-6G (neutrophils, DCs) (clone 1A8, BD Biosciences) to quantitate granulocytes. AccuCount Fluorescent Particles (12.5 µl of 5.2 µm size, Spherotech, Inc.) were added to determine absolute counts (cells/µl). Whole blood was lysed after staining with FACS lysis buffer (BD Biosciences) and analyzed on a FACSVantage (BD Biosciences). Cells were gated for forward and side scatter and dead cells (a very small fraction) were excluded. Ten thousand events (excluding beads) were obtained. Granulocytes (Ly-6G^+^, CD14^−^, F4-80^−^) were quantified. In some samples, absolute counts of granulocytes were measured using volumetric/flow-rate calibration [40], [41]. Stained and lysed whole blood was analyzed for a fixed amount of time that resulted in identical amounts of volume to be analyzed. The total number of granulocytes were then calculated based on the volume of the 50 µl of blood analyzed. All samples from each experiment were analyzed at the same time to avoid possible variations in flow rates that could occur at different days, temperatures, or relative humidities.

### Statistics

The effect of hypogravity and/or radiation on the number of granulocytes counted and serum levels of corticosterone were determined. The results from groups of 10 mice were averaged and comparisons were analyzed by 2-way ANOVA (StatPlus). The effect of hypogravity and/or radiation on the number of bacteria counted in blood or lung was analyzed using the Friedman ANOVA (non-parametric) with Wilcoxon signed-rank post-hoc analysis with Bonferroni correction (Prism). Morbidity scores (Table 1) were calculated daily and animals were considered morbid if their score increased by 3 points or remained 2 points elevated for 24 hrs or death of the animal. Statistical significance between groups was measured using the log rank test (Xlstat) on Kaplan-Meier analyses.

## Results

Three different strains of mice were used. ICR mice are an outbred strain initiated in 1948 from Swiss mice. C3H/HeN mice are inbred and have no known defects or polymorphisms that impair DNA repair or the response to ionizing radiation. Balb/c mice have 2 different polymorphisms in DNA-dependent protein kinase (DNA-PKcs) that mediates non-homologous end joining, which results in decreased but not absent function [42], [43], [44]. Balb/c mice are considered a radiation sensitive strain and are used as a representative surrogate for humans with similar increased sensitivity to radiation effects due to reduced repair enzyme function [45]. To accurately measure the effect of the hindlimb suspension model of microgravity and stress and SPE-like radiation on the ability of different strains of mice to effectively clear a challenge with bacteria previously demonstrated to cause infections in astronauts, hindlimb suspended and/or irradiated mice were exposed 5 days later to *Pseudomonas aeruginosa* delivered intraperitoneally (IP) or *Klebsiella pneumoniae* delivered by inhalation. Mice had blood drawn at various times before and after bacterial challenge and were followed for a total of 5 days after bacterial challenge (Figure 1). Blood from 3 and 5 days and homogenized lung at day 5 (from *Klebsiella* infected mice) post bacterial challenge was plated on agar in serial dilutions to quantitate the number of CFUs present and signs of morbidity (Table 1) were followed.

**Figure 2:**
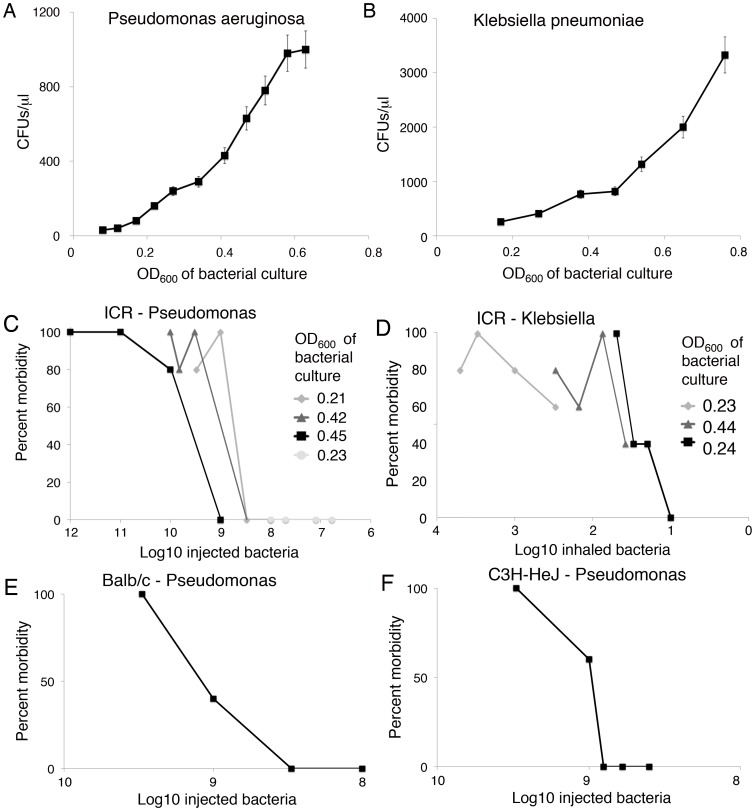
Determination of bacterial challenge dose. The association between the OD_600_ of the bacterial culture and the number of CFUs/µl was determined for *Pseudomonas aeruginosa* (A) and *Klebsiella pneumoniae* (B) bacteria by plating dilutions of culture and quantitating colonies. The OD_600_ quantitation of CFU/µl was then used to determine the amount of bacteria that untreated mice could control with minimal morbidity in ICR mice with *Pseudomonas aeruginosa* delivered IP (C) and *Klebsiella pneumoniae* delivered by inhalation (D). The numbers of CFUs of *Klebsiella pneumoniae* were found to be the same for Balb/c and C3H/HeN mice as for ICR mice. The maximal number of CFUs of *Pseudomonas aeruginosa* that resulted in the ability of Balb/c (E) and C3H/HeN (F) mice to control the infection with minimal morbidity was determined.

The maximal number of CFUs of each bacterium in each mouse strain that an untreated control mouse could control with minimal morbidity was calculated. As CFUs cannot be calculated in real time, the OD of the bacterial cultures at 600 nm was used and correlated with CFUs/µl (Figure 2A and B). Increasing amounts of bacteria were delivered to untreated mice (Pseudomonas by the IP route and Klebsiella by inhalation) and morbidity was followed over 5 days (Figure 2C–E). The number of inhaled *Klebsiella pneumoniae* bacteria needed was determined to be the same for all mouse strains in initial studies. To further decrease variation in the number of CFUs delivered, challenges were performed using bacterial cultures within a narrow OD_600_ range (0.40–0.45 for *Pseudomonas*, 0.5–0.6 for *Klebsiella*). The maximal doses of each bacterium that allowed clearance and minimal morbidity were used for each strain of mouse and each bacterium.

During travel outside the Earth's magnetic field, an astronaut would be subjected to variable and extended durations of time in microgravity before exposure to a potentially harmful SPE. To model this, we initially placed mice in hindlimb suspension for 0, 2, or 5 days prior to irradiation. Similar findings were found for each condition, but increased morbidity and weight loss in the absence of bacterial challenge was observed with extending the time in suspension. As the addition of time in suspension prior to irradiation did not change the results, irradiation and hindlimb suspension were performed at the same time.

**Figure 3:**
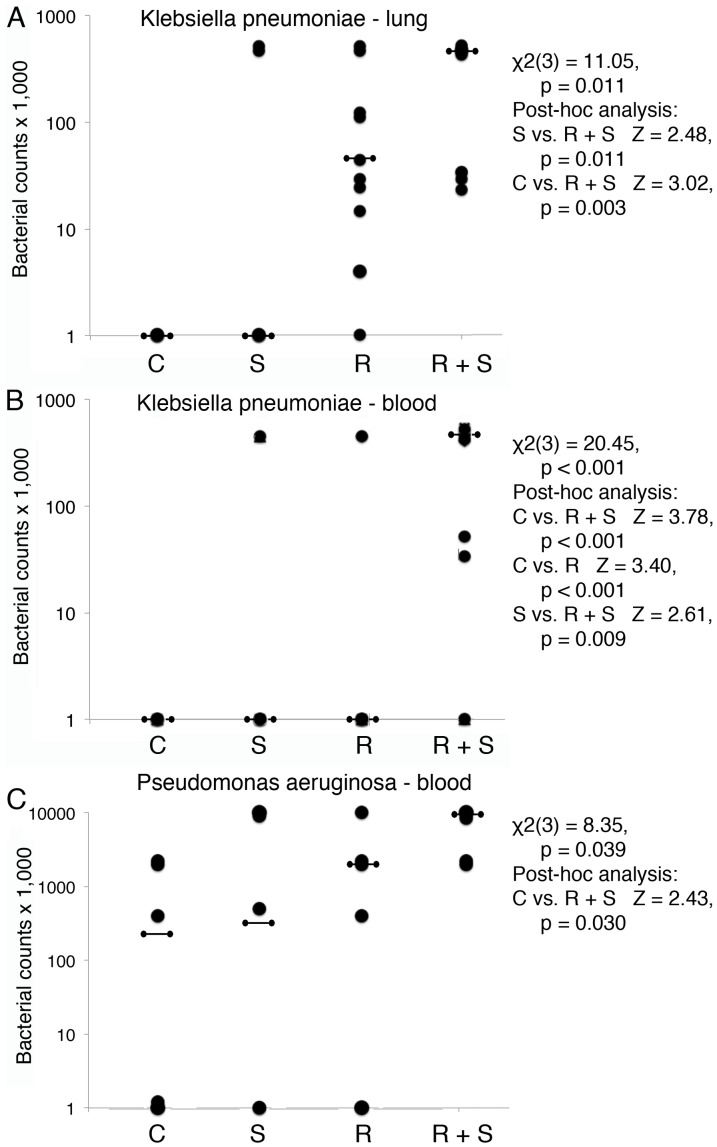
Proton radiation and hindlimb suspension of ICR mice impairs their ability to clear a lung challenge with *Klebsiella pneumoniae* or a peritoneal challenge with *Pseudomonas aeruginosa*. Groups of 10 female ICR mice per treatment group were not treated, irradiated with 2/or placed in hindlimb suspension. Five days later, mice were exposed to *Klebsiella pneumoniae* by inhalation and followed. Lung tissue (A) and whole blood (B) was plated to quantitate CFUs of bacteria 5 days later. Similarly treated mice were delivered *Pseudomonas aeruginosa* IP and followed. After 5 days, bacteria in blood was quantitated (C). C =  control, S =  hindlimb suspended, R =  irradiated, and R+S =  irradiated and suspended. Median values are indicated by a barbell. The Friedman ANOVA, degrees of freedom in parentheses, with Wilcoxon signed-rank post-hoc analysis with Bonferroni correction was used to test statistical significance.

ICR mice were hindlimb suspended and/or irradiated with 2 Gy of proton radiation and challenged with *Klebsiella pneumoniae* by inhalation. Mice were followed daily for signs of systemic and pulmonary infection. CFUs of bacteria were quantitated in lung tissue and blood 5 days after challenge. Mice that received both radiation and hindlimb suspension demonstrated a significant inability to clear bacteria from the lungs or bloodstream (Figure 3A–B). Similarly treated ICR mice were challenged with *Pseudomonas aeruginosa* IP. CFUs of bacteria were quantitated in the blood 5 days after challenge. Radiation and hindlimb suspended mice demonstrated a significant inability to clear bacteria from the bloodstream (Figure 3C). All mice were placed in radiation enclosures for the same amount of time and non-suspended mice were individually caged, similar to hindlimb suspended mice.

**Figure 4:**
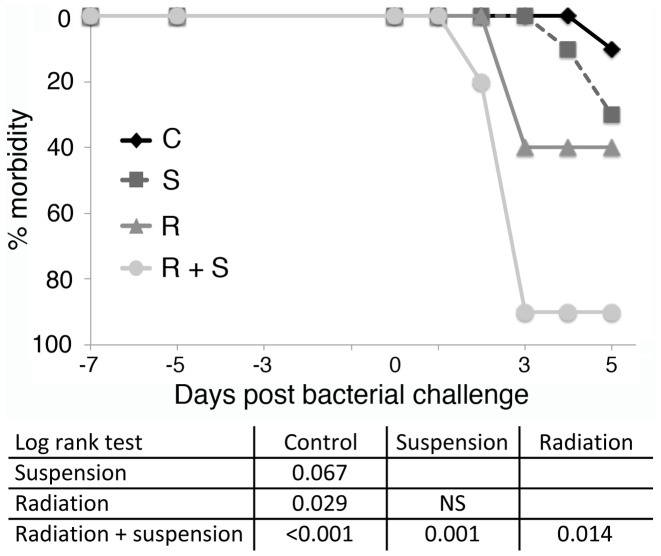
C3H/HeN mice treated with hindlimb suspension and 2 Gy of proton irradiation fail to control a challenge with *Pseudomonas aeruginosa* bacteria. Groups of 10 C3H/HeN mice per treatment group were not treated, proton irradiated (2 Gy), and/or placed in hindlimb suspension. Five days later, mice were exposed to *Pseudomonas aeruginosa* by intraperitoneal injection and followed for 5 days. Morbidity scores (Table 1) were calculated daily and animals were considered morbid if their score increased by 3 points or remained 2 points elevated for 24 hrs. C =  control, S =  hindlimb suspended, R =  irradiated, and R+S =  irradiated and suspended. Statistical significance was measured by log rank analysis of Kaplan-Meier curves and expressed in the table as column versus rows.

Proton irradiated and hindlimb suspended C3H/HeN mice received *Pseudomonas aeruginosa* IP and were followed for signs of systemic infection. Bacteremia was monitored 3 and 5 days post infection and morbidity as defined in Table 1 was followed. Mice were euthanized if their morbidity score increased by 3 points or remained elevated by 2 points for greater than 24 hrs. Radiation alone resulted in a statistically significant increase in morbidity and the combination of radiation and hindlimb suspension had at least an additive effect on increasing morbidity and often a greater than additive effect (Figure 4). Comparisons between experiments demonstrated differences in the total number of irradiated and suspended animals that had morbid events or did not clear bacteremia. This was due to the requirement for a less accurate surrogate for CFUs/µl in the challenge dose (OD_600_ of the culture), since CFU calculation required overnight culture for accurate measurement. The relative increase in morbidity or lack of clearance of bacteremia was consistent across experiments. When expressed as relative increase in morbidity and replicate experiments were analyzed together, hindlimb suspension and radiation led to statistically significant increases in morbidity.

**Figure 5:**
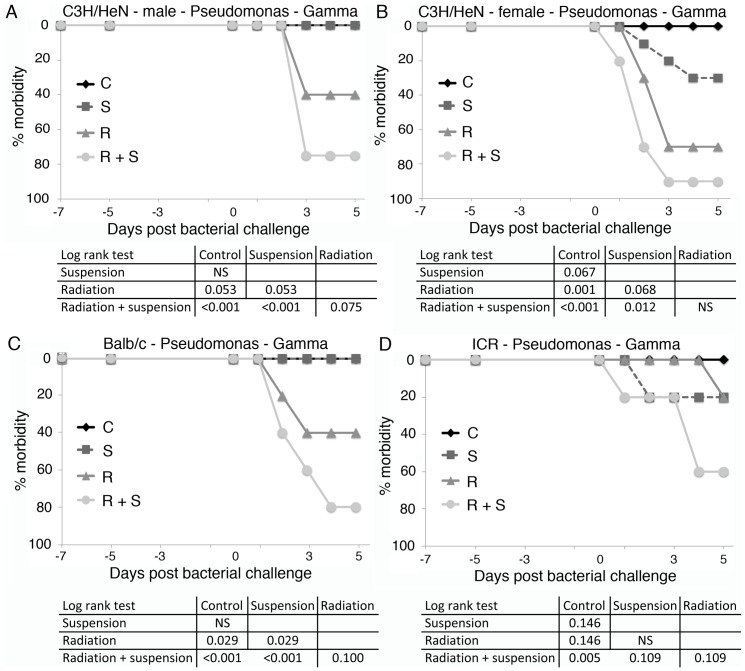
Gamma irradiated mice treated with hindlimb suspension fail to control a challenge with *Pseudomonas aeruginosa*. Groups of 10 C3H/HeN male (A) and female (B), Balb/c female (C) and ICR female (D) mice were gamma irradiated (2 Gy) and/or placed in hindlimb suspension. Five days later, mice were exposed to *Pseudomonas aeruginosa* by intraperitoneal injection and followed for 5 days. Morbidity scores (Table 1) were calculated daily and animals were considered morbid if their score increased by 3 points or remained elevated 2 points for 24 hrs. C =  control, S =  hindlimb suspended, R =  irradiated, and R+S =  irradiated and suspended. Statistical significance was measured by log rank analysis of Kaplan-Meier curves and expressed in the table as column versus rows.

**Figure 6:**
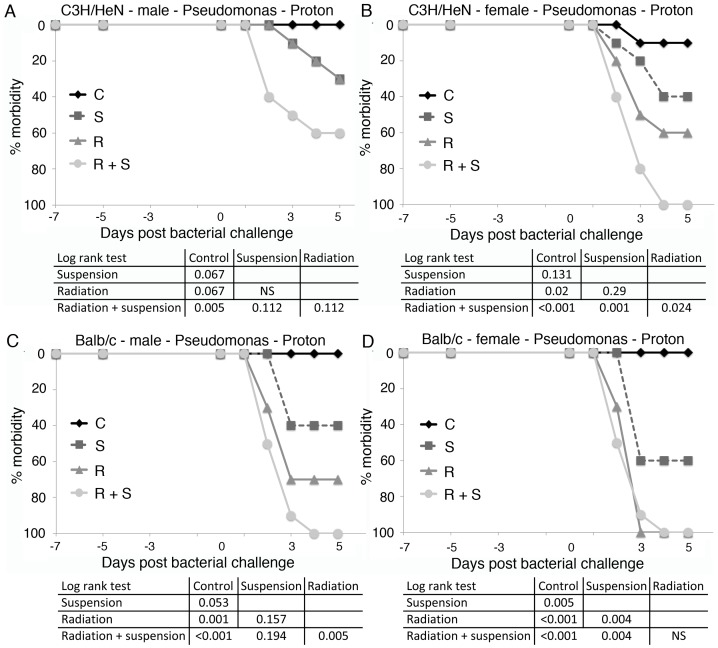
Proton irradiated mice treated with hindlimb suspension fail to control a challenge with *Pseudomonas aeruginosa*. Groups of 10 C3H/HeN male (A) and female (B) and Balb/c male (C) and female (D) mice were proton irradiated (2 Gy) and/or placed in hindlimb suspension. Five days later, mice were exposed to *Pseudomonas aeruginosa* by intraperitoneal injection and followed for 5 days. Morbidity scores (Table 1) were calculated daily and animals were considered morbid if their score increased by 3 points or remained elevated 2 points for 24 hrs. C =  control, S =  hindlimb suspended, R =  irradiated, and R+S =  irradiated and suspended. Statistical significance was measured by log rank analysis of Kaplan-Meier curves and expressed in the table as column versus rows.

**Figure 7:**
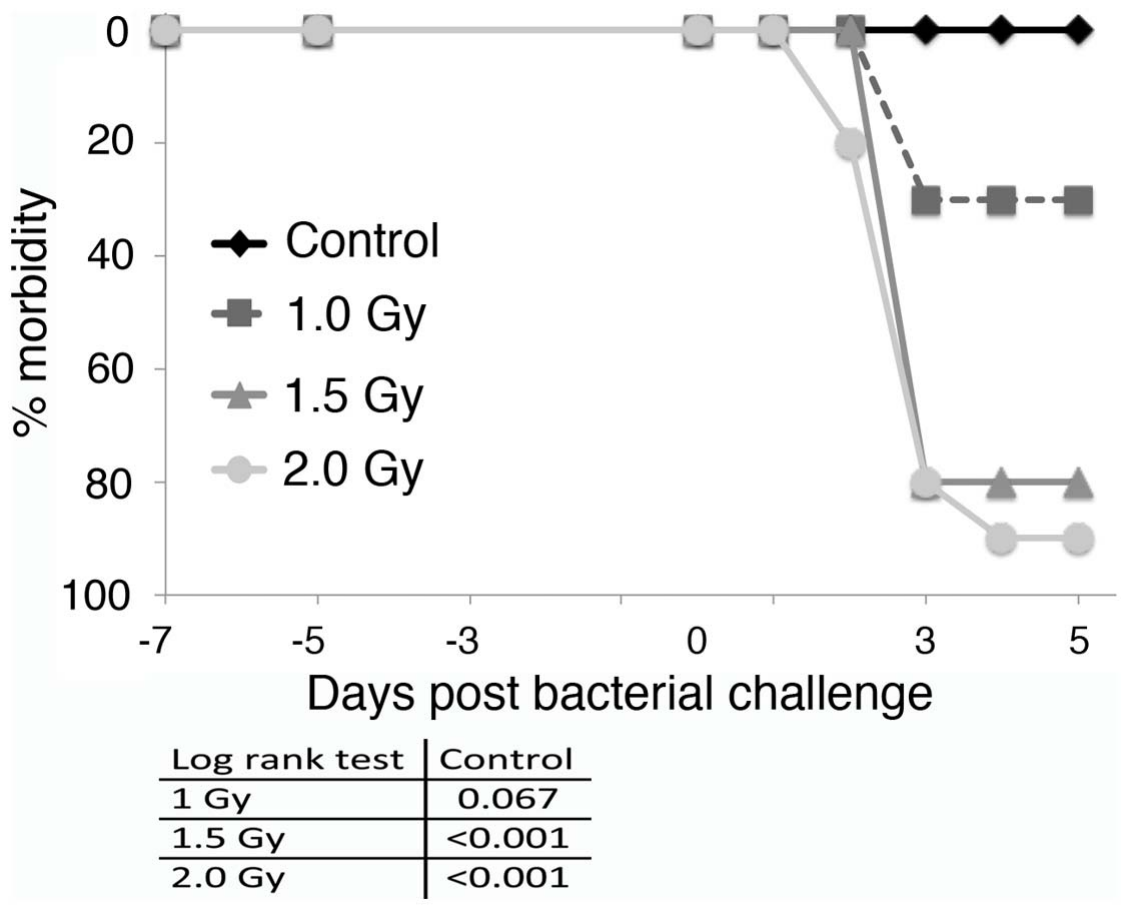
Dose response of gamma radiation with hindlimb suspension on the ability to control a challenge with *Pseudomonas aeruginosa*. Groups of 10 C3H/HeN female mice were gamma irradiated with 2.0, 1.5, or 1.0 Gy and placed in hindlimb suspension. Five days later, mice were exposed to *Pseudomonas aeruginosa* by intraperitoneal injection and followed for 5 days. Morbidity scores (Table 1) were calculated daily and animals were considered morbid if their score increased by 3 points or remained elevated 2 points for 24 hrs. Statistical significance was measured by log rank analysis of Kaplan-Meier curves.

Gamma radiation, as a more commonly used type of ionizing radiation, was employed as a reference and to investigate relative biologic effect (RBE). Two Gy of gamma radiation yielded similar amounts of increased morbidity and an at least additive effect when combined with hindlimb suspension (Figure 5A–B). Differences in immune reactivity and susceptibility to infectious diseases between males and females is well described (reviewed in [40], [41], [46], [47], [48]). To determine whether sex affects the response to hindlimb suspension and gamma or proton radiation, both male and female mice were analyzed. Similar relative levels of morbidity were observed after challenge with *Pseudomonas* bacteria in male and female mice (Figure 5A–B, Figure 6). The effect of smaller amounts of gamma irradiation in combination with hindlimb suspension on *Pseudomonas aeruginosa* challenge of female C3H/HeN mice was determined. One and a half Gy of total body radiation impaired the ability of mice to control the bacterial challenge similar to that observed for 2 Gy (Figure 7). Interestingly, little difference in the amount of morbidity was observed between 2.0 and 1.5 Gy. One Gy of gamma radiation gave similar results as mice treated with hindlimb suspension alone (Figure 5B). All mice, including controls, were placed in irradiation chambers for the same amount of time.

As similar results were obtained with both bacterial challenges, data for *Pseudomonas aeruginosa* will be presented as representative of both systems. Balb/c mice, a radiation sensitive strain with polymorphisms in its DNA-PKcs genes, were irradiated and/or hindlimb suspended and challenged IP with *Pseudomonas aeruginosa*. Similar to C3H/HeN (Figure 5 and 6) and ICR (Figure 4 and 5) mice, increased morbidity (Figure 5 and 6) was observed. Similar data was obtained for males and females and gamma and proton radiation (Figure 5 and 6). The relative increases in morbidity were similar for all 3 strains of mice, suggesting that the polymorphisms in DNA-PKcs in Balb/c mice did not significantly affect the response to a bacterial challenge when the mice were irradiated and/or exposed to hindlimb suspension.

**Figure 8:**
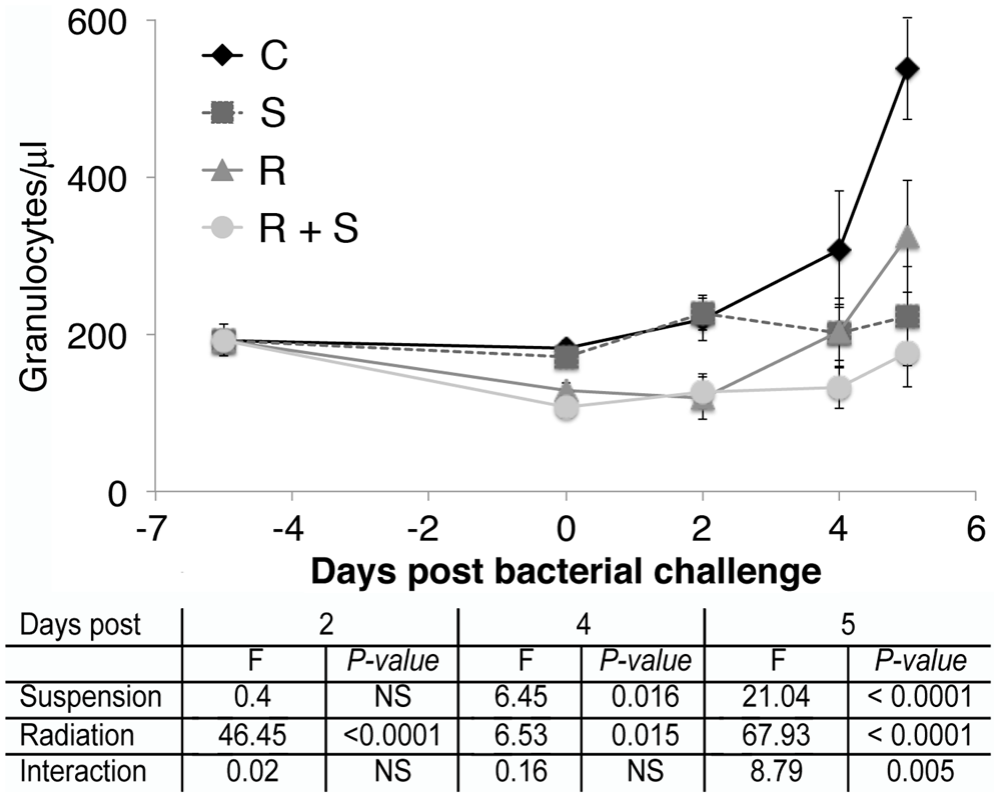
Hindlimb suspension impairs the increase in peripheral blood granulocytes during systemic bacteremia. C3H/HeN mice were hindlimb suspended and irradiated with 2 Gy of gamma radiation. Five days later, mice were challenged with *Pseudomonas aeruginosa* by intraperitoneal injection. The number of granulocytes, Ly-6G^high^, CD14^−^, and F4-80^−^ in peripheral blood was calculated using AccuCount fluorescent particles at the indicated time points before and after bacterial challenge. Data from 10 mice in each group were averaged and error bars are standard error of the mean. C =  control, S =  hindlimb suspended, R =  irradiated, and R+S =  irradiated and suspended. Statistical significance of the effect of hindlimb suspension and irradiation and the interaction between them was calculated by 2-way ANOVA for the indicated days post bacterial challenge.

Peripheral blood granulocyte counts were determined in C3H/HeN mice challenged with *Pseudomonas aeruginosa* at time points prior to hindlimb suspension and irradiation and before and after bacterial challenge (Figure 8). A reduction in the peripheral blood granulocyte counts was observed after radiation similar to that described previously [26], [49], [50]. After bacterial challenge, both the control animals and the radiation only treated mice increased peripheral blood granulocyte counts, while the mice treated with hindlimb suspension, with or without radiation, failed to elevate blood granulocyte counts. Similar blunting of the peripheral blood granulocyte response to systemic infection by hindlimb suspension was observed in Balb/c and ICR mice (Figure S1).

**Figure 9:**
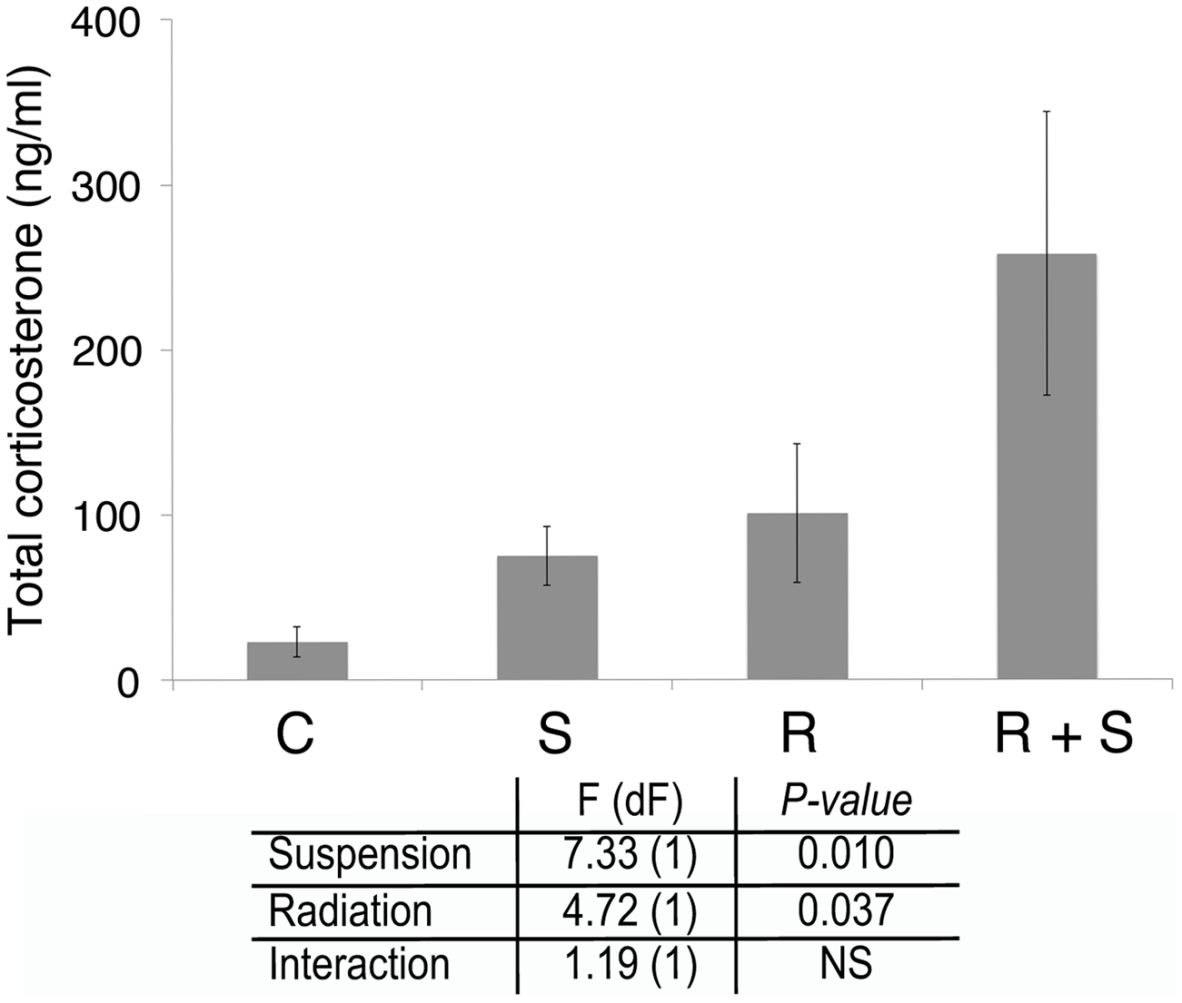
Hindlimb suspension and/or 2 Gy of proton radiation increases serum total corticosterone levels. Groups of 10 C3H/HeN female mice per treatment group were placed in hindlimb suspension and/or proton irradiated (2 Gy). Five days later, serum was obtained and analyzed for total corticosterone by ELISA. Data from 10 mice in each group were averaged and error bars are standard error of the mean. C =  control, S =  hindlimb suspended, R =  irradiated, and R+S =  irradiated and suspended. Statistical significance of the effect of hindlimb suspension and irradiation and the interaction between them was calculated by 2-way ANOVA.

The observation that hindlimb suspension impaired the granulocyte response to bacterial infection is unexpected. A major physiological strain during spaceflight that is modeled during hindlimb suspension is stress that can negatively regulate immune function. Cortisol or corticosterone is induced during various types of stress and impairs both innate and acquired immune function (reviewed in [51]). Total corticosterone was measured in mice 5 days after beginning hindlimb suspension and/or irradiation just prior to bacterial challenge. Suspended and irradiated animals had an increase in total corticosterone and animals that received both hindlimb suspension and 2 Gy of radiation had a further elevated level of free corticosterone (Figure 9).

## Discussion

Future space missions will involve extended journeys outside the protection of the Earth's magnetic field. During extravehicular activities without this protection, an astronaut could receive 32 Gy of radiation to the skin and 2 Gy to the bone marrow during a strong SPE [27], [29]. Ideally, avoidance of exposure to an SPE would be the ideal countermeasure, but they are unpredictable and it is expected that an astronaut will have less than one hour notice, or no notice at all, of an incoming SPE [52]. The combined effects of prolonged space flight and intermittent exposure to solar radiation could render astronauts susceptible to bacterial or other infections. We used the hindlimb unloading model of microgravity and proton radiation with an energy spectrum similar to that expected during an SPE, as well as reference gamma radiation, to determine the effect of these space conditions on controlling bacterial infection. In both inbred and outbred strains of mice, and using 2 different bacteria previously demonstrated to cause infections in astronauts during spaceflight, we demonstrate a significant impairment in the ability to control infection with a massive increase in morbidity. Either hindlimb suspension or radiation alone results in increased morbidity and reduced ability to clear bacteria and the combination is at least additive and often greater than additive. Formal RBE calculations were not performed, as no significant difference in the effect of gamma versus proton radiation was evident, yielding an RBE of 1.

Radiation (2 Gy) either gamma or proton results in a significant decrease in granulocyte counts in peripheral blood (Figure 8 and supplemental Figure 1) and other organs [26], [49], [50]. In the setting of a bacterial infection, this results in a reduction of effector cells capable of controlling the infection. Studies of humans receiving cancer chemotherapy have indicated that the risk of infection increases as the peripheral granulocyte count decreases and patients are given empiric antibiotics if they develop a fever or signs of infection and their granulocyte count is below 500/µl (reviewed in [53]). The etiology of the impairment to control bacterial infection by hindlimb suspension is less clear. Previous studies have observed a range of immune defects induced by the hindlimb suspension model of microgravity, including thymic involution similar to that observed with space flight [54], reduction in the ability of phagocytes to kill bacteria and generate superoxide radicals [55], reductions in T cell activation and cytokine production, and multiple impairments to immune effector function [23], [56], [57], [58], [59], [60]. In our studies, hindlimb suspension inhibited the ability of systemic bacterial infection to increase peripheral blood granulocyte counts (Figure 8 and supplemental Figure 1) in the setting of a significant increase in total corticosterone levels (Figure 9), which was associated with an impaired ability to control the infection. It is noteworthy that radiation alone led to a similar increase in corticosterone and irradiated mice, while starting at a lower level of blood granulocytes, were still able to increase them in the setting of systemic bacterial infection (Figure 8 and supplemental Figure 1). The ability of hindlimb suspension to impair the physiologic granulocyte response to bacterial infection is a new and unexpected observation. Studies are ongoing to determine the mechanisms of the abnormal granulocyte response induced by hindlimb unloading.

Studies have indicated a reduction in the ability of hindlimb unloaded mice to control bacterial infections. Suspended mice given subcutaneous *Pseudomonas aeruginosa* had increased mortality and organ bacterial load and an increase in corticosterone [25]. Hindlimb suspended mice given IP *Klebsiella pneumoniae* similarly had an increase in mortality and bacterial organ loads [24]. Our data agree with and extends these data to include the additional space risk of SPE-like radiation. We observed a significant increase in corticosterone levels for hindlimb suspension, which is in agreement with most studies of hindlimb suspension [2], [58], [61], [62]. Two Gy of radiation also increased corticosterone levels and a greater than additive increase was observed when mice were treated with both. The studies of systemic *Klebsiella pneumoniae* and *Pseudomonas aeruginosa* gave mice an LD_50_ of bacteria [24], [25], which allowed small changes in immune function to result in significant increases in mortality above the expected 50%. We used doses of bacteria that control mice could consistently control; thus, any increase in morbidity or decrease in clearance of bacteria is directly applicable to astronaut exposures during space flight.

There are multiple spaceflight conditions that impair immune function, including microgravity, confinement and situational stress, altered food intake and circadian rhythms, and exposure to space radiation. The greatly increased morbidity associated with hindlimb suspension and SPE-like radiation suggests that astronauts on extended missions outside of the Earth's magnetic field are at increased risk of potentially serious infections due to the impairment of immune function. Countermeasures to protect astronauts from infections due to the at least additive effects of SPE-like radiation and microgravity are currently being investigated.

## Supporting Information

Figure S1 **Hindlimb suspension impairs the increase in peripheral blood granulocytes during systemic bacteremia.** C3H/HeN (A), ICR (B and C), and Balb/c (D and E) were hindlimb suspended and irradiated with 2 Gy of protons or gamma radiation, as indicated. Five days later, mice were challenged with *Pseudomonas aeruginosa* by intraperitoneal injection. The number of granulocytes, Ly-6G^high^, CD14^−^, and F4-80^−^ in peripheral blood was calculated on 5 days after bacterial challenge. Data from 3–10 mice in each group were averaged and error bars are standard error of the mean. C =  control, S =  hindlimb suspended, R =  irradiated, and R+S =  irradiated and suspended. Statistical significance of the effect of hindlimb suspension and irradiation and the interaction between them was calculated by 2-way ANOVA.
(TIF)
