# Supplementary figures and images for: Hindlimb Suspension and SPE-Like Radiation Impairs Clearance of Bacterial Infections
Source: PLoS One. 2014 Jan 15;9(1):e85665. doi: 10.1371/journal.pone.0085665 (PMC3893249; doi:10.1371/journal.pone.0085665)

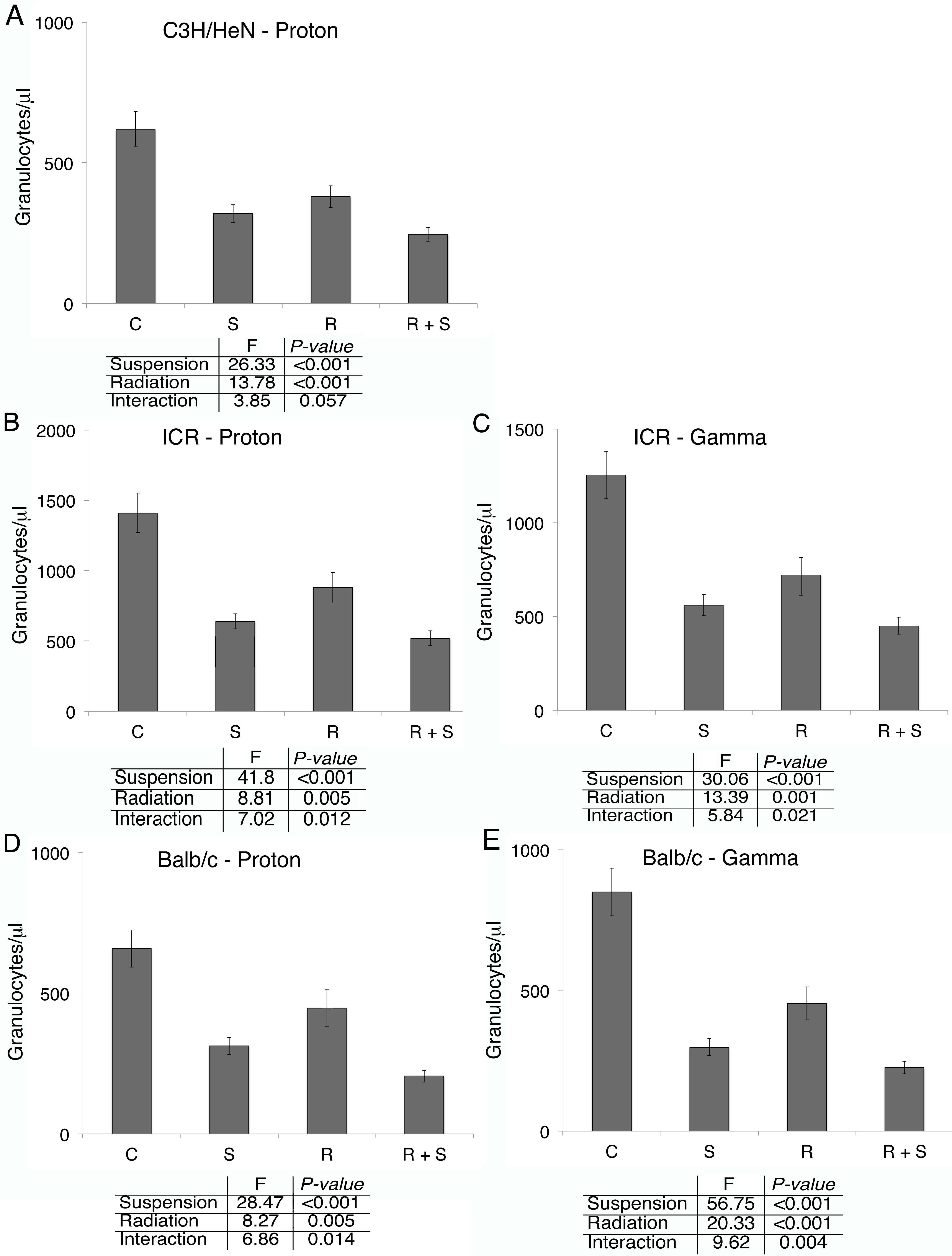

Supplement: Figure S1 — Hindlimb suspension impairs the increase in peripheral blood granulocytes during systemic bacteremia. C3H/HeN (A), ICR (B and C), and Balb/c (D and E) were hindlimb suspended and irradiated with 2 Gy of protons or gamma radiation, as indicated. Five days later, mice were challenged with Pseudomonas aeruginosa by intraperitoneal injection. The number of granulocytes, Ly-6Ghigh, CD14−, and F4-80− in peripheral blood was calculated on 5 days after bacterial challenge. Data from 3–10 mice in each group were averaged and error bars are standard error of the mean. C = control, S = hindlimb suspended, R = irradiated, and R+S = irradiated and suspended. Statistical significance of the effect of hindlimb suspension and irradiation and the interaction between them was calculated by 2-way ANOVA. (TIF) [file pone.0085665.s001.tif]
